# Prognostic value of measurement of myocardial extracellular volume using dual-energy CT in heart failure with preserved ejection fraction

**DOI:** 10.1038/s41598-024-58271-9

**Published:** 2024-03-29

**Authors:** Ying Jiang, Jiaqi Ye, Yang Yang, Ying Zhang, Xiaoyun Yan, Wenhui Qiang, Haixiao Chen, Shuang Xu, Lei Zhou, Rongxing Qi, Qing Zhang

**Affiliations:** 1 Department of General Practice, The Second Affiliated Hospital of Nantong University, Shengli Road No.666, Nantong, 226001 China; 2 Department of Radiology, The Second Affiliated Hospital of Nantong University, Shengli Road No.666, Nantong, 226001 China; 3 Department of Cardiology, The First Affiliated Hospital of Nanjing Medical University, Nanjing, 210029 China

**Keywords:** Cardiology, Cardiovascular diseases

## Abstract

Diffuse myocardial fibrosis is associated with adverse outcomes in heart failure with preserved ejection fraction (HFpEF). Dual-energy CT (DECT) can noninvasively assess myocardial fibrosis by quantification of extracellular volume (ECV) fraction. This study evaluated the association between ECV measured by DECT and clinical outcomes in patients with HFpEF. 125 hospitalized HFpEF patients were enrolled in this retrospective cohort study. ECV was measured using DECT with late iodine enhancement. The composite endpoint was defined as HFpEF hospitalization and all-cause mortality during the follow-up. During the median follow-up of 10.4 months, 34 patients (27.20%) experienced the composite outcomes, including 5 deaths; and 29 HFpEF hospitalizations. The higher DECT-ECV group had higher rates of composite outcomes than the low ECV group (log-rank *X*^2^ = 6.818, *P* = 0.033). In multivariate Cox regression analysis, the ECV (*HR* 1.17, 95% *CI* 1.06–1.30, *P* = 0.001) and NT-pro BNP (*HR* 2.83, 95% *CI* 1.16–6.88,* P* = 0.022) were independent risk factors for the adverse outcomes. Myocardial ECV measured using DECT was an independent risk factor for adverse outcomes in patients with HFpEF.

## Introduction

Diffuse myocardial fibrosis may play a key role in the development and worsening of heart failure, and it is associated with adverse outcomes^1^. Imaging techniques allow noninvasive approaches to the surrogate assessment of myocardial fibrosis, becoming attractive tools for identifying cardiac fibrosis, risk stratification, and treatment management.

Cardiac computed tomography (CCT) has become an effective application for myocardial fibrosis assessment over the past decade. Through late iodine enhancement (LIE), CCT can estimate extracellular volume fraction (ECV), a new imaging biomarker for interstitial expansion due to myocardial fibrosis^2^, which has been validated against Cardiac magnetic resonance (CMR)^3,4^ and histopathology^5^. Advanced dual-energy CT (DECT) allows myocardial tissue characterization with different kV levels based on the attenuation identity of different materials at different energies^6^. This technology allows for reducing imaging artifacts and increasing the contrast-to-noise ratio to improve LIE image quality^7^. Compared with conventional single-energy CT, ECV measured by DECT has a greater correlation with CMR-ECV^8^.

Heart failure with preserved ejection fraction (HFpEF) is an important heart failure phenotype. The prevalence of HFpEF relative to heart failure with a reduced ejection fraction (HFrEF) has risen steadily and the ratio of HFpEF/HFrEF was reversal from 41/59 in the decade 1985–1994 to 56/44 in the decade 2005–2014 in the Framingham study^9^. HFpEF is a multiorgan syndrome resulting from comorbidities like metabolic risk, arterial hypertension, and renal insufficiency, which predispose to systemic inflammation and coronary microvascular endothelial dysfunction, resulting in left ventricular remodeling and diastolic dysfunction^10^. Myocardial fibrosis may contribute to myocardial stiffness and diastolic dysfunction in HFpEF^11,12^, and ECV measured using CMR has been confirmed associated with adverse outcomes in HFpEF^13–15^. Compared with CMR, assessment of myocardial fibrosis using DECT has the potential advantages of easy acquisition and not being limited by the presence of implantable electronic devices^7^. It is unclear whether ECV quantified by DECT could also be associated with poor outcomes in HFpEF. Thus, this study measured ECV using DECT and investigated the association between ECV and the clinical outcomes of patients with HFpEF.

## Methods

### Study population

This was a single-center study and we retrospectively reviewed the hospitalized HFpEF patients who underwent DECT examination in the Second Affiliated Hospital of Nantong University. From January 2019 to December 2020, 125 patients were enrolled in the study. HFpEF, defined according to the 2016 European Society for Cardiology guidelines for the diagnosis and treatment of acute and chronic heart failure^16^. The inclusion criteria were: (1) presence of signs or symptoms of heart failure and left ventricular ejection fraction (LVEF) ≥ 50%. (2) B-type natriuretic peptide (BNP) ≥ 100 pg/ml or N-terminal pro-BNP (NT-pro BNP) ≥ 300 pg/ml. (3) New York Heart Association (NYHA) grade II or higher. (4) Patients underwent hematocrit and NT-pro-BNP measurements were performed within a 24-h interval of DECT. Exclusive criteria included: (1) Contraindication to iodinated contrast agents. (2) Patients with an estimated glomerular filtration rate (eGFR) < 30 ml/min/1.73 m^2^. (3) Acute coronary syndrome or with a history of myocardial infarction, significant valvular heart disease (i.e. greater than moderate left-sided valve disease); known or suspected hypertrophic/infiltrative cardiomyopathy or constrictive pericarditis, amyloidosis, and adult congenital heart disease. (4) Previous percutaneous coronary intervention, or coronary artery bypass grafting.

### Ethics approval

The study complied with the principles of the Declaration of Helsinki and was approved by the committee of the institutional review board at the Second Affiliated Hospital of Nantong University (No. 2020KN094). All patients signed the informed consent forms.

### Baseline characteristics and DECT scan protocol

We collected baseline characteristics, clinical data from the hospital medical records, including age, sex, body mass index (BMI), smoking, drinking, hypertension, diabetes, atrial fibrillation and blood pressure. Fasting venous blood was collected, and biochemistry tests were performed in the second morning after the patients were admitted. Hemoglobin, fasting plasma glucose (FPG), glycated hemoglobin A_1c_ (HbA_1_c), serum creatinine (Scr), triglycerides (TG), low-density lipoprotein cholesterol (LDL-C) and NT-pro BNP level were detected.

All patients were examined with a third-generation dual-source CT (Somatom Force, Siemens Healthcare, Forchheim, Germany). Before the examination, if the patient's heart rate exceeded 75 bpm, 25–50 mg metoprolol (AstraZeneca Pharmaceuticals Co., Ltd.) would be given to slow the heart rate. A bolus of 50 ml iopromide (Ultravist 370, Bayer Pharmaceuticals & Healthcare Co., Ltd.) was then injected into the antecubital vein at a flow rate of 4.5–5.0 ml/s, followed by 30 ml of saline as a bolus chaser injected at the same flow rate.

During the CCT scan, the following procedures included: a prospective electrocardiography (ECG)-gated calcium score acquisition, a prospective ECG-gated coronary CT angiography (CCTA) and a delay DECT scan. The scan ranged from 1 cm below the tracheal bifurcation to the diaphragmatic level of heart. The scanning parameters included: A-tube voltage 100 kV, B-tube sn140 kV, automatic tube current modulation technology, a 192 × 0.6 mm collimation, and 0.15 pitch factor. The acquisition phase was 65–80% of the R-R interval. The DECT scanning was conducted 7 min later with the same parameters and scan range for the calcification score acquisition. All images were reconstructed with a matrix 512 × 512, slice thickness of 0.6 mm, interval of 0.4 mm and convolution kernel (Qr36). The effective radiation dose of CT was calculated by multiplying the dose-length product by a conversion factor of 0.014.

### DECT data post processing and ECV measurement

**Figure 1:**
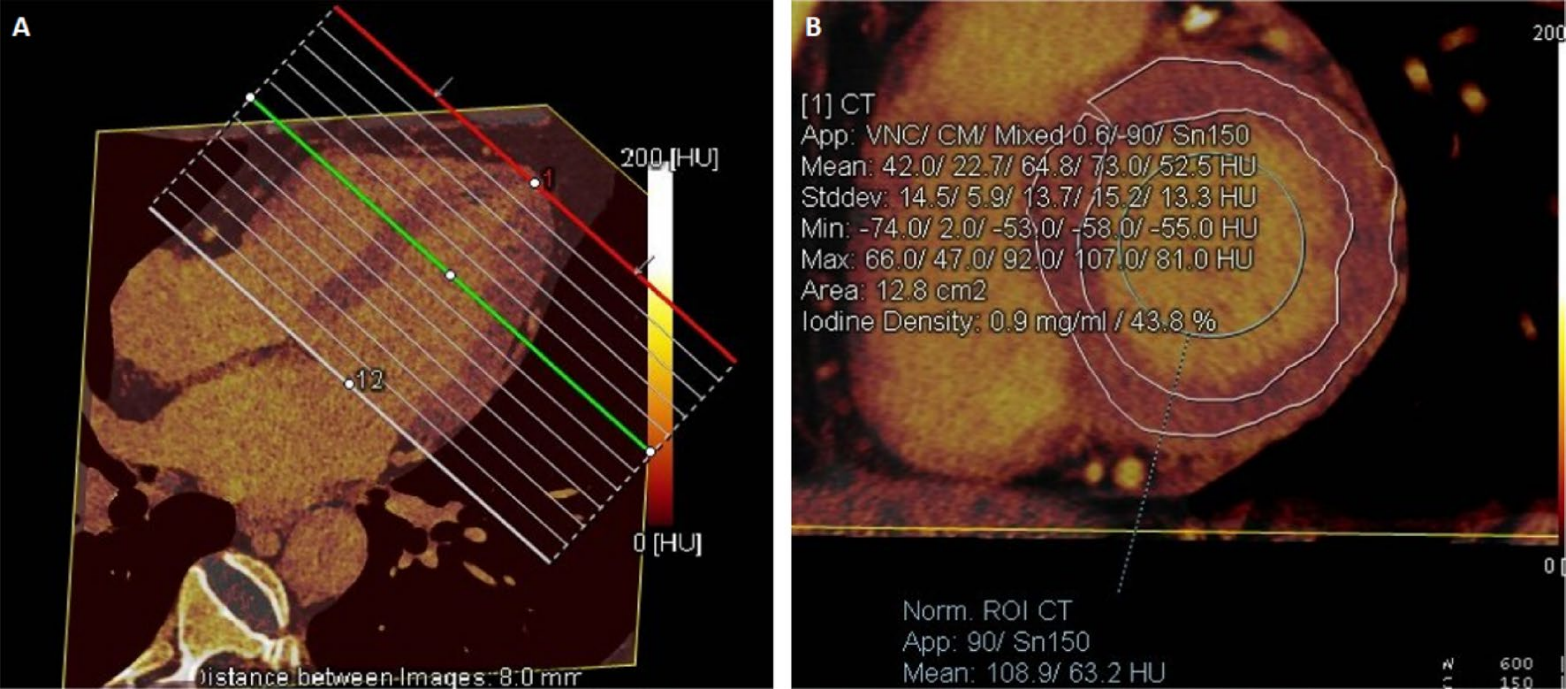
Iodine map shows the measurement of ECV with low value. (**A**) The iodine map were reconstructed in a short axis view with an 8-mm thickness from the base to apex. (**B**) A 75-year-old man with HFpEF, myocardial iodine/blood iodine is 43.8% for the base left ventricle, 42.5% for the middle left ventricle, and 36.5% for the apes left ventricle. The serum hematocrit level is 44.2%. The mean ECV of the left ventricle base is calculated as 24.44%, and the mean ECV of the middle, apex, and whole left ventricle is calculated as 23.72%, 20.2%, and 23.11%.

**Figure 2:**
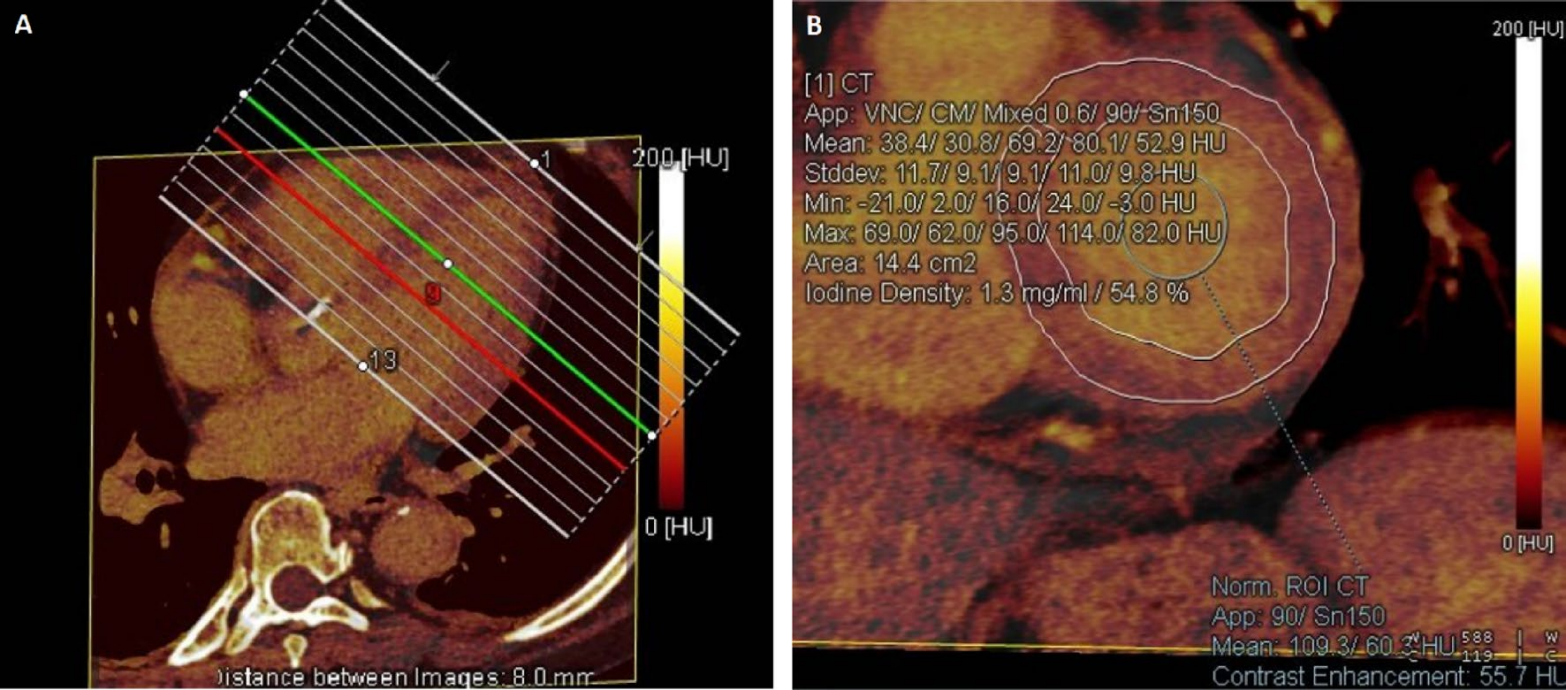
Iodine map shows the measurement of ECV with high value. (**A**) The iodine maps were reconstructed in a short axis view with an 8-mm thickness from the base to apex. (**B**) A 74-year-old woman with HFpEF, myocardial iodine/blood iodine is 54.8% for the base left ventricle, 51.5% for the middle left ventricle, and 59.1% for the apes left ventricle. The serum hematocrit level is 32.4%. The mean ECV of the left ventricle base is calculated as 37.04%, and the mean ECV of the middle, apex, and whole left ventricle is calculated as 34.81%, 39.95%, and 36.93%.

The DECT post-processing was performed on a workstation (Syngo via, VB20, Siemens Medical Solutions, Forchheim, Germany). The iodine maps were constructed by the ‘heart PBV’ software, based on the material decomposition method, which displayed the distribution of iodine in the left ventricle. The iodine maps were reconstructed in a short axis view with an 8-mm thickness from the base to apex of the heart without any gaps. According to the 16-segment of left ventricular myocardium^17^, the regions of interest (ROIs) were manually conservatively drawn in each segment to avoid the periphery of the myocardium. The ROI in the blood pool with a size over 100 mm^2^ excluded the papillary muscles and focal delayed myocardial enhancement. The evaluation of ECV was completed by two experienced observers. DECT-ECV was calculated as follows: ECV (%) = (iodine density of myocardium/iodine density of blood pool) × (1 − hematocrit level) × 100%, the iodine density of myocardium is the myocardial iodine density of delay enhancement, and iodine density of the blood pool is the enhanced iodine density of the left ventricular blood pool (Figs. 1, 2).

### Echocardiographic measurement

All study participants underwent transthoracic echocardiography by an experienced echocardiographer using Philips IE33 ultrasound scanner with S5-1 transducer. Cardiac structure and function were assessed following recommendations of the American Society of Echocardiography and European Association of Echocardiography guidelines^18,19^. Briefly, echocardiographic parameters including left ventricular volume, ejection fraction, and diastolic functions. Left ventricular end-diastolic volume (LVEDV), left ventricular end-systolic volume (LVESV), and LVEF were derived from Simpson’s biplane method. Left atrial volume (LAV) was measured using the area-length method from the apical two- and four-chamber views at ventricular end-systole. This was then divided by body surface area to obtain the left atrial volume index (LAVI). Peak early diastolic trans-mitral flow velocity (E-wave) and early diastolic mitral annular velocity (eʹ) to estimate LV filling pressures were assessed by Doppler echocardiography. These were then averaged to calculate E/eʹ ratio.

### Follow-up and endpoint ascertainment

The study period lasted for 12 months. The majority of patients were followed by reviewing their electronic medical records, while telephone interviews were conducted with some participants in special circumstances (such as contacting family members if the patient passed away). The composite endpoint in the current study was defined as HFpEF hospitalization and all-cause death.

### Statistical analysis

All statistical analyses were performed by SPSS 22.0 software. The continuous variables were assessed through the Shapiro–Wilk test and presented as either mean and standard deviation (SD) for normal distribution or median and interquartile range for non-normal distribution. The categorical variables were presented as numbers and percentages. To compare baseline characteristics of patients with or without composite outcomes, the Mann–Whitney U test was used for non-normally distributed continuous variables, the two-tailed t-test for normally distributed continuous variables, and the χ^2^ test for categorical variables. Bland–Altman analysis was run to evaluate intra-observer and inter-observer agreements for the ECV measurements. The correlation between variables and ECV was evaluated using the Pearson or Spearman correlation. Linear regression analysis was run to identify the factors associated with ECV.

Patients were divided into three groups according to the ECV. Kaplan–Meier curves were constructed and the log-rank test was used to measure differences in the outcome events of these groups. Risk factors of the endpoint were analyzed using both univariate and multivariate-adjusted Cox regression analyses. Hazard ratios (HRs) and their corresponding 95% confidence intervals (CIs) were presented as well. *P* < 0.05 was recognized as a statistical difference.

## Results

### Study characteristics

All 125 patients completed the DECT examination and none of them lost during the follow-up, with the mean age of 63.98 ± 12.14 years. They were categorized as with or without the composite outcomes. During the median follow-up of 10.4 months, there were 34 patients (27.20%) experienced the composite outcomes, including 5 deaths; and 29 HFpEF hospitalizations. The demographic and clinical characteristics are presented in Table 1. Compared with the patients without adverse outcomes, patients with adverse outcomes had higher age, ECV and NT-pro BNP level (*P* < 0.05).

**Table 1:** Baseline characteristics of HFpEF patients.

	All patients (n = 125)	With events (n = 34)	Without events (n = 91)	*P* value
Male^a^	56 (44.80)	13 (38.24)	43 (47.25)	0.367
Age (years)^b^	63.98 ± 12.14	67.21 ± 9.76	62.78 ± 12.75	0.042
BMI (kg/m^2^)^c^	24.61 (22.49, 26.70)	25.81 (22.42, 27.65)	24.49 (22.49, 26.22)	0.293
Smoking^a^	23 (18.40)	8 (23.53)	15 (16.48)	0.366
Drinking^a^	28 (22.40)	9 (26.47)	19 (20.88)	0.505
Hypertension^a^	81 (64.80)	25 (73.53)	56 (61.54)	0.212
Diabetes^a^	44 (35.20)	14 (41.18)	30 (32.97)	0.392
Atrial fibrillation^a^	41 (32.80)	15 (44.12)	27 (29.67)	0.128
Heart rate (bpm)^b^	71.90 ± 10.35	72.76 ± 9.71	71.58 ± 10.61	0.339
Systolic BP (mmHg)^b^	147.30 ± 23.42	152.32 ± 25.68	145.43 ± 22.37	0.144
Diastolic BP (mmHg)^b^	85.18 ± 14.67	88.21 ± 15.09	84.04 ± 14.42	0.159
Hemoglobin (g/l)^b^	130.99 ± 18.30	126.44 ± 17.72	132.69 ± 18.31	0.089
FPG (mmol/l)^c^	5.58 (4.99, 6.32)	5.62 (4.90, 6.55)	5.54 (5.01, 6.32)	0.929
HbA_1c_ (mmol/l)^c^	6.10 (5.67, 7.10)	5.80 (5.28, 6.80)	6.20 (5.73, 7.10)	0.101
Scr (μmol/l)^c^	69.80 (58.85, 80.70)	67.05 (56.75, 84.93)	70.10 (59.00, 79.80)	0.861
eGFR (ml/min/1.73 m^2^)^c^	88.43 (67.64, 102.54)	85.97 (61.00, 97.03)	90.33 (69.38, 105.86)	0.187
TG (mmol/l)^c^	1.43 (0.99, 2.26)	1.43 (0.98, 2.02)	1.42 (0.99, 2.28)	0.914
LDL-C (mmol/l)^c^	2.60 (1.99, 3.30)	2.43 (1.75, 3.13)	2.65 (2.06, 3.38)	0.101
NT-pro BNP (pg/ml)^c^	1179.00 (618.45, 2026.50)	1484.50 (908.35, 2947.00)	1102.00 (671.90, 1714.00)	0.033
LVEF (%)^c^	57.00 (54.00, 62.00)	56.00 (52.75, 62.00)	58.00 (54.00, 63.00)	0.115
LVEDV (ml)^b^	90.66 ± 15.90	93.74 ± 15.19	89.51 ± 16.08	0.293
LVESV (ml)^b^	38.10 ± 12.87	39.40 ± 12.31	37.61 ± 13.12	0.990
LVMI (g/m^2^)^b^	104.17 ± 28.16	106.40 ± 29.82	103.33 ± 27.63	0.545
LAV (ml)^b^	57.95 ± 17.84	59.79 ± 17.17	57.26 ± 18.13	0.946
LAVI (ml/m^2^)^b^	35.20 ± 8.94	36.29 ± 9.89	34.79 ± 8.58	0.391
E wave (cm/s)^b^	81.63 ± 14.74	83.74 ± 15.35	80.85 ± 14.51	0.955
eʹ (cm/s)^b^	5.93 ± 1.26	6.12 ± 1.16	5.86 ± 1.30	0.581
E/eʹ (cm/s)^b^	14.25 ± 3.40	14.85 ± 3.92	14.02 ± 3.17	0.234
Hematocrit level (%)^b^	40.14 ± 4.91	39.51 ± 5.44	40.37 ± 4.71	0.389
ECV (%)^b^	29.22 ± 3.79	31.03 ± 3.57	28.54 ± 3.66	0.001
Medication during hospital				
SGLT-2i^a^	34 (27.20)	12 (35.29)	22 (24.18)	0.214
ARNI/ACEI/ARB^a^	77 (61.60)	23 (67.65)	54 (59.34)	0.395
Aldosterone antagonist^a^	79 (63.20)	22 (64.71)	57 (62.64)	0.831
β-Blockers^a^	55 (44.40)	16 (47.06)	39 (42.86)	0.674

*BMI* body mass index, *BP* blood pressure, *FPG* fasting plasma glucose, *HbA*_*1c*_ glycated hemoglobin A_1c_, *Scr* serum creatinine, *eGFR* the glomerular filtration rate, *TG* triglycerides, *LDL-C* low-density lipoprotein cholesterol, *NT-pro BNP* N-terminal pro-B-type natriuretic peptide, *LVEF* left ventricular ejection fraction, *LVEDV* left ventricular end-diastolic volume, *LVESV* left ventricular end-systolic volume, *LVMI* left ventricular mass index, *LAV* left atrial volume, *LAVI* left atrial volume index, *ECV* extracellular volume, *SGLT-2i* sodium-glucose cotransporter2 inhibitor, *ARNI/ACEI/ARB* angiotensin receptor-neprilysin inhibitor or angiotensin-converting enzyme inhibitors or angiotensin II receptor blockers.

Categorical variables are expressed as ‘^a^’as number (percentages). Continuous variables are presented as ‘^b^’as mean (standard deviation) or as ‘^c^’as median (interquartile range).

### ECV assessment with DECT

**Figure 3:**
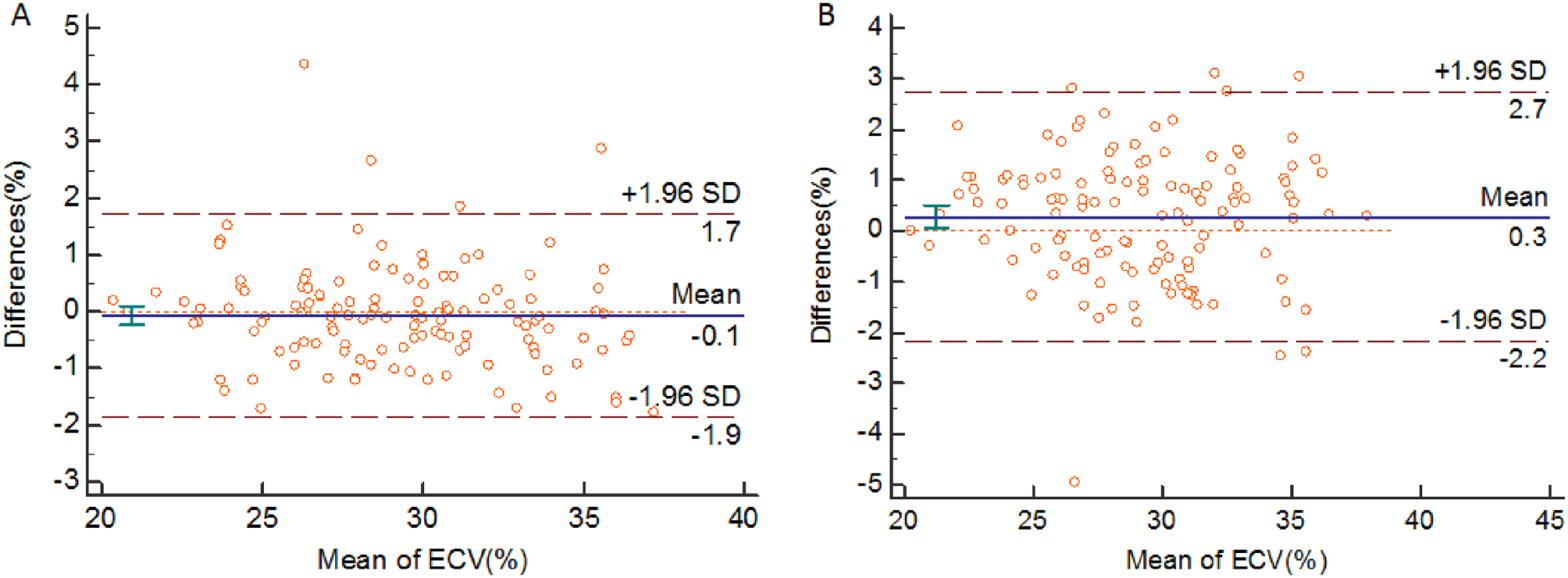
Bland–Altman analysis of ECV for intra-observer (**A**) and inter-observer (**B**).

Mean ECV was 29.22 ± 3.79. In Bland–Altman analysis (Fig. 3), the mean intra-observer difference was − 0.070% (95% LOA, − 1.854% to 1.715%), and the mean inter-observer difference was 0.278% (95% LOA, − 2.176% to 2.732%).

### Associations of clinical variables with ECV

The correlation analysis in Table 2 showed that age (γ = 0.192,* P* = 0.032), LVEF (γ =  − 0.259,* P* = 0.003), LVMI (γ = 0.331,* P* = 0.000), LAVI (γ = 0.253,* P* = 0.004), E/eʹ (γ = 0.265,* P* = 0.003) were associated with ECV. In the multivariate linear regression model, the LVEF (standardized β =  − 0.211,* P* = 0.007), LVMI (standardized β = 0.331,* P* = 0.000), LAVI (standardized β = 0.177,* P* = 0.024), E/eʹ (standardized β = 0.300,* P* = 0.000) were still correlated with ECV.

**Table 2:** Association between clinical variables and ECV in HFpEF.

Variables	γ	*P* value
Sex	− 0.017	0.847
Age*	0.192	0.032
BMI	− 0.068	0.449
Smoking	0.120	0.184
Drinking	− 0.012	0.890
Hypertension	0.110	0.223
Diabetes	0.097	0.281
Atrial fibrillation	0.022	0.805
Heart rate*	0.036	0.687
Systolic BP*	0.119	0.187
Diastolic BP*	0.045	0.615
Hemoglobin*	− 0.087	0.333
FPG	− 0.002	0.986
HbA_1c_	0.021	0.837
Scr	0.094	0.297
TG	0.085	0.343
LDL-C	− 0.158	0.079
NT-pro BNP	0.008	0.929
LVEF	− 0.259	0.003
LVEDV*	0.123	0.172
LVESV*	0.114	0.204
LVMI*	0.331	0.000
LAV*	0.147	0.102
LAVI*	0.253	0.004
E wave*	0.160	0.075
eʹ*	0.069	0.447
E/eʹ*	0.265	0.003

*BMI* body mass index, *BP* blood pressure, *FPG* fasting plasma glucose, *HbA*_*1c*_ glycated hemoglobin A_1c_, *Scr* serum creatinine, *TG* triglycerides, *LDL-C* low-density lipoprotein cholesterol, *NT-pro BNP* N-terminal pro-B-type natriuretic peptide, *LVEF* left ventricular ejection fraction, *LVEDV* left ventricular end-diastolic volume, *LVESV* left ventricular end-systolic volume, *LVMI* left ventricular mass index, *LAV* left atrial volume, *LAVI* left atrial volume index.

*Variables which exhibited using Pearson correlation.

### Prognostic value of ECV in HFpEF

**Figure 4:**
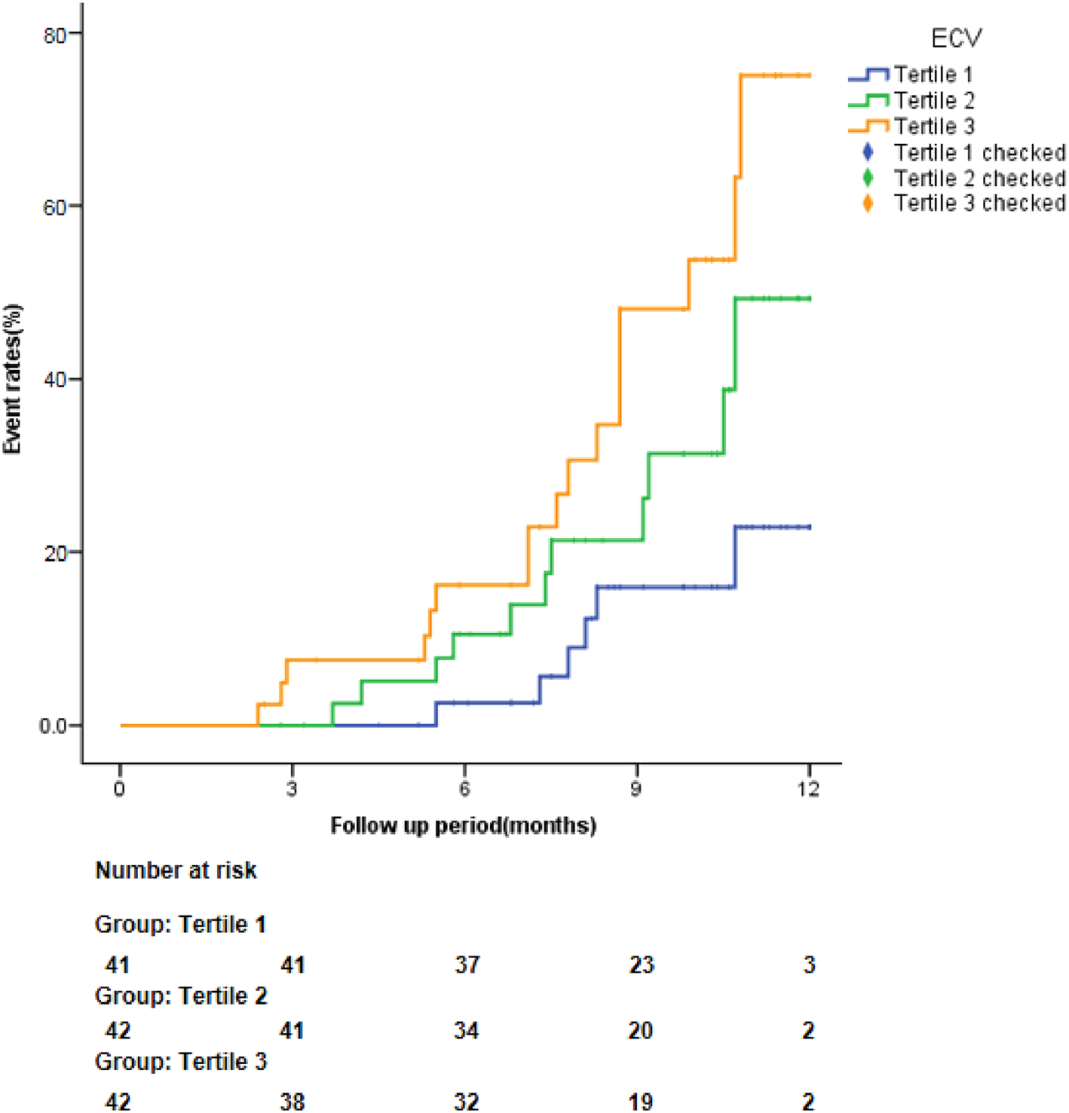
Cumulative incidence curve for the composite endpoint according to ECV.

During the median follow-up period of 10.4 months, 34 patients (27.20%) experienced the composite endpoint. According to the tertiles of ECV, patients were divided into tertile 1 (41 cases, 20.47–27.30%), tertile 2 (42 cases, 27.31–30.83%) and tertile 3 (42 cases, 30.84–37.0%). Kaplan–Meier analysis in Fig. 4 showed the higher level of ECV fraction was a significant predictor of composite outcomes (log–rank *X*^2^ = 6.818, *P* = 0.033).

In the multivariate Cox regression analysis, after adjusting for age, hemoglobin, the ECV (*HR* 1.17, 95% *CI* 1.06–1.30, *P* = 0.001) and NT-pro BNP (*HR* 2.83, 95% *CI* 1.16–6.88, *P* = 0.022) were independent risk factors for the composite endpoint in Table 3.

**Table 3:** Risk factors analysis for the composite endpoint.

Variables	Univariate		Multivariate	
	HR (95% CI)	*P* value	HR (95% CI)	*P* value
Sex	0.65 (0.33, 1.31)	0.229		
Age	1.03 (1.00, 1.06)	0.079	1.01(0.98,1.04)	0.491
Smoking	1.81 (0.82, 4.01)	0.145		
Hypertension	1.51 (0.71, 3.24)	0.289		
Diabetes	1.33 (0.67, 2.64)	0.412		
Atrial fibrillation	1.749 (0.89, 3.44)	0.106		
Hemoglobin	0.983 (0.96, 1.03)	0.098	0.986 (0.97, 1.01)	0.183
NT-pro BNP	2.90 (1.20, 7.00)	0.018	2.83 (1.16, 6.88)	0.022
LVEF	0.96 (0.90, 1.01)	0.135		
ECV	1.18 (1.08, 1.30)	0.001	1.17 (1.06–1.30)	0.001

*CI* confidence interval, *NT-pro BNP* N-terminal pro-B-type natriuretic peptide, *LVEF* left ventricular ejection fraction, *ECV* extracellular volume.

## Discussion

In this study, we investigated ECV measured using DECT with late iodine enhancement in patients with HFpEF. And we demonstrated the significant association between ECV and clinical outcomes in HFpEF.

Heart failure is the final stage of cardiovascular disease, and nearly 50% of cases are caused by HFpEF^20^. Myocardial fibrosis is a significant pathological process in HFpEF. When there is an injury to the myocardium, the cardiac fibroblasts become active and differentiate into myofibroblasts, whose secretome causes alterations in the extracellular processing of fibrillary collagen facilitating the excessive accumulation of collagen fibers, and the extracellular matrix deposited, leading to myocardial fibrosis, reducing cardiac compliance, myocardial stiffness, ventricular diastolic dysfunction and heart failure^1,21^. Kanagala et al.^22^ found that the extent of myocardial fibrosis is significantly associated with an increased risk of hospitalization and death in patients with HFpEF. Therefore, the detection of structural myocardial fibrosis is of major prognostic value.

ECV assesses myocardial fibrosis, and noninvasive cardiac imaging modalities such as CMR, and CCT, are used to evaluate ECV. Although CMR is currently the great effective imaging method for the clinical assessment of ECV^23^, it has several limitations, such as limited scan slices, and contraindication, in patients with claustrophobia or implanted pacemaker^24^. Compared with CMR, DECT has the potential advantages of ease of acquisition and not being limited by the presence of implantable electronic devices^7^. Recent studies have validated that ECV calculated with DECT has a great correlation with CMR-ECV^4,25^, and DECT-ECV measured using iodine-density method with delayed enhancement is more accurate than the subtraction method using single-energy CT^26^, with CMR as the reference. Therefore, we used the iodine-density method derived from DECT to measure ECV. In this study, we found patients with higher ECV fraction had more adverse events than those with lower ECV. This is consistent with previous evidence that myocardial fibrosis is associated with poor outcomes in HFpEF.

In line with previous studies^27^, we also confirmed that NT-pro BNP was an important biomarker of the prognosis in HFpEF. NT-pro BNP is a circulating cardiac biomarker of myocardial stretch and biomechanical stress, and is advised in HFpEF guidelines^16^. Furthermore, NT-pro BNP specifies cardiac dysfunction and is robustly associated with adverse outcomes^28,29^. The levels of NT-pro BNP are increased in HFpEF and mirror the severity of cardiac morphological and functional abnormalities, such as LV hypertrophy, fibrosis and diastolic dysfunction^30^. Schelbert et al.^14^ have shown that ECV measured with CMR were strongly associated with log-transformed BNP levels in a sub-cohort of patients with HFpEF. However, our study did not show that NT-pro BNP was associated with ECV. Wu et al. also found NT-pro BNP level was unable to differentiate the degree of fibrosis well, and that it was associated with heart failure symptoms^31^. In addition to the limited small sample size, another possible reason for this is that NT-pro BNP is not just a marker of volume overload and could be influenced by the character and duration of loading conditions^32^. Factors that impact NT-pro BNP should also be taken into consideration, including atrial fibrillation, kidney disease, diabetic ketosis, obesity and increasing age^29^.

We found that ECV measured with DECT was associated with echocardiographic parameters about systolic and diastolic dysfunction. In our study, ECV was negatively correlated with LVEF and positively correlated with LVMI, LAVI, E/eʹ. Previous studies have confirmed the relationship between these parameters and ECV derived from CMR. Wang et al. found a negative correlation between LVEF and ECV^4^. Kanagala et al. found ECV was significantly associated with E/eʹ, left ventricular mass/volume, maximal LAVI^22^. This implies there is a structure–function relationship between ECV changes and segmental myocardial function.

### Study limitations

This study has several limitations. First, this single center retrospective study enrolled a small population of HFpEF patients and set a short follow-up period, which would not avoid recall bias and residual confounders. In the future, prospective multicenter clinical trials with larger samples are necessary to investigate the characteristics of DECT-ECV in the subgroups of different types of heart failure and to analyze its relationship with long-term prognosis. Second, the ECV was available only at baseline, not measured dynamically during the follow-up. Therefore, any changes in ECV may have occurred in response to the therapeutic management of HFpEF are unknown and need further exploration.

## Conclusion

In summary, the findings of this study confirm that myocardial ECV measured using DECT was associated with the composite outcomes of patients with HFpEF, which may be an independent risk factor for predicting prognosis in this population.

## Data Availability

The datasets used and/or analysed during the current study are available from the corresponding author on reasonable request.

## References

[1] Ravassa S (2023). Cardiac fibrosis in heart failure: Focus on non-invasive diagnosis and emerging therapeutic strategies. Mol. Aspects Med..

[2] Treibel TA (2017). Automatic quantification of the myocardial extracellular volume by cardiac computed tomography: Synthetic ECV by CCT. J. Cardiovasc. Comput. Tomogr..

[3] Ohta Y (2020). Investigation of myocardial extracellular volume fraction in heart failure patients using iodine map with rapid-kV switching dual-energy CT: Segmental comparison with MRI T1 mapping. J. Cardiovasc. Comput. Tomogr..

[4] Wang R (2018). Extracellular volume quantitation using dual-energy CT in patients with heart failure: Comparison with 3T cardiac MR. Int. J. Cardiol..

[5] Bandula S (2013). Measurement of myocardial extracellular volume fraction by using equilibrium contrast-enhanced CT: Validation against histologic findings. Radiology.

[6] Patino M (2016). Material separation using dual-energy CT: Current and emerging applications. Radiographics.

[7] Gupta S, Ge Y, Singh A, Gräni C, Kwong RY (2021). Multimodality imaging assessment of myocardial fibrosis. JACC Cardiovasc. Imaging.

[8] Kumar V (2019). Estimation of myocardial fibrosis in humans with dual energy CT. J. Cardiovasc. Comput. Tomogr..

[9] Vasan RS (2018). Epidemiology of left ventricular systolic dysfunction and heart failure in the Framingham study: An echocardiographic study over 3 decades. JACC Cardiovasc. Imaging.

[10] Shah SJ (2016). Phenotype-specific treatment of heart failure with preserved ejection fraction: A multiorgan roadmap. Circulation.

[11] Rommel KP (2016). Extracellular volume fraction for characterization of patients with heart failure and preserved ejection fraction. J. Am. Coll. Cardiol..

[12] Mohammed SF (2015). Coronary microvascular rarefaction and myocardial fibrosis in heart failure with preserved ejection fraction. Circulation.

[13] Golukhova E, Bulaeva N, Alexandrova S, Gromova O, Berdibekov B (2022). Prognostic value of characterizing myocardial tissue by cardiac MRI with T1 mapping in HFpEF patients: A systematic review and meta-analysis. J. Clin. Med..

[14] Schelbert EB (2017). Temporal relation between myocardial fibrosis and heart failure with preserved ejection fraction: Association with baseline disease severity and subsequent outcome. JAMA Cardiol..

[15] Rush CJ (2021). Prevalence of coronary artery disease and coronary microvascular dysfunction in patients with heart failure with preserved ejection fraction. JAMA Cardiol..

[16] Ponikowski P (2016). 2016 ESC Guidelines for the diagnosis and treatment of acute and chronic heart failure: The Task Force for the diagnosis and treatment of acute and chronic heart failure of the European Society of Cardiology (ESC) developed with the special contribution of the Heart Failure Association (HFA) of the ESC. Eur. Heart J..

[17] Cerqueira MD (2002). Standardized myocardial segmentation and nomenclature for tomographic imaging of the heart. A statement for healthcare professionals from the Cardiac Imaging Committee of the Council on Clinical Cardiology of the American Heart Association. Int. J. Cardiovasc. Imaging.

[18] Lang RM (2015). Recommendations for cardiac chamber quantification by echocardiography in adults: An update from the American Society of Echocardiography and the European Association of Cardiovascular Imaging. J. Am. Soc. Echocardiogr..

[19] Nagueh SF (2016). Recommendations for the evaluation of left ventricular diastolic function by echocardiography: An update from the American Society of Echocardiography and the European Association of Cardiovascular Imaging. J. Am. Soc. Echocardiogr..

[20] Redfield MM, Borlaug BA (2023). Heart failure with preserved ejection fraction: A review. JAMA.

[21] de Boer RA (2019). Towards better definition, quantification and treatment of fibrosis in heart failure. A scientific roadmap by the Committee of Translational Research of the Heart Failure Association (HFA) of the European Society of Cardiology. Eur. J. Heart Fail..

[22] Kanagala P (2019). Relationship between focal and diffuse fibrosis assessed by CMR and clinical outcomes in heart failure with preserved ejection fraction. JACC Cardiovasc. Imaging.

[23] Ambale-Venkatesh B, Lima JA (2015). Cardiac MRI: A central prognostic tool in myocardial fibrosis. Nat. Rev. Cardiol..

[24] Suzuki M (2021). Prognostic impact of myocardial extracellular volume fraction assessment using dual-energy computed tomography in patients treated with aortic valve replacement for severe aortic stenosis. J. Am. Heart Assoc..

[25] Lee HJ (2016). Myocardial extracellular volume fraction with dual-energy equilibrium contrast-enhanced cardiac CT in nonischemic cardiomyopathy: A prospective comparison with cardiac MR imaging. Radiology.

[26] Emoto T (2021). Myocardial extracellular volume quantification using cardiac computed tomography: A comparison of the dual-energy iodine method and the standard subtraction method. Acad. Radiol..

[27] Myhre PL (2018). Association of natriuretic peptides with cardiovascular prognosis in heart failure with preserved ejection fraction: Secondary analysis of the TOPCAT randomized clinical trial. JAMA Cardiol..

[28] Berezin AE, Berezin AA (2023). Biomarkers in heart failure: From research to clinical practice. Ann. Lab. Med..

[29] Morfino P (2022). Biomarkers of HFpEF: Natriuretic peptides, high-sensitivity troponins and beyond. J. Cardiovasc. Dev. Dis..

[30] Islam MN, Chowdhury MS, Paul GK, Debnath RC, Shakil SS (2019). Association of diastolic dysfunction with N-terminal pro-B-type natriuretic peptide level in heart failure patients with preserved ejection fraction. Mymensingh Med. J..

[31] Wu CK, Su MM, Wu YF, Hwang JJ, Lin LY (2018). Combination of plasma biomarkers and clinical data for the detection of myocardial fibrosis or aggravation of heart failure symptoms in heart failure with preserved ejection fraction patients. J. Clin. Med..

[32] Schulz O (2011). Influence of acute and chronic myocardial loading conditions, function, structural changes and extracardiac factors on NT-proBNP in asymptomatic patients with preserved ejection fraction. Clin. Res. Cardiol..

